# Resistome of carbapenem- and colistin-resistant *Klebsiella pneumoniae* clinical isolates

**DOI:** 10.1371/journal.pone.0198526

**Published:** 2018-06-08

**Authors:** Sara Lomonaco, Matthew A. Crawford, Christine Lascols, Ruth E. Timme, Kevin Anderson, David R. Hodge, Debra J. Fisher, Segaran P. Pillai, Stephen A. Morse, Erum Khan, Molly A. Hughes, Marc W. Allard, Shashi K. Sharma

**Affiliations:** 1 Center for Food Safety and Applied Nutrition, U.S. Food & Drug Administration, College Park, Maryland, United States of America; 2 Division of Infectious Diseases & International Health, University of Virginia, Charlottesville, Virginia, United States of America; 3 National Center for Emerging and Zoonotic Infectious Diseases, Centers for Disease Control and Prevention, Atlanta, Georgia, United States of America; 4 IHRC, Inc., Atlanta, Georgia, United States of America; 5 Science and Technology Directorate, U.S. Department of Homeland Security, Washington, DC, United States of America; 6 Office of the Commissioner, U.S. Food & Drug Administration, Silver Spring, Maryland, United States of America; 7 Division of Select Agents, Centers for Disease Control and Prevention, Atlanta, Georgia, United States of America; 8 Department of Pathology and Laboratory Medicine, Aga Khan University, Karachi, Pakistan; Cornell University, UNITED STATES

## Abstract

The emergence and dissemination of carbapenemases, bacterial enzymes able to inactivate most β-lactam antibiotics, in *Enterobacteriaceae* is of increasing concern. The concurrent spread of resistance against colistin, an antibiotic of last resort, further compounds this challenge further. Whole-genome sequencing (WGS) can play a significant role in the rapid and accurate detection/characterization of existing and emergent resistance determinants, an essential aspect of public health surveillance and response activities to combat the spread of antimicrobial resistant bacteria. In the current study, WGS data was used to characterize the genomic content of antimicrobial resistance genes, including those encoding carbapenemases, in 10 multidrug-resistant *Klebsiella pneumoniae* isolates from Pakistan. These clinical isolates represented five sequence types: ST11 (n = 3 isolates), ST14 (n = 3), ST15 (n = 1), ST101 (n = 2), and ST307 (n = 1). Resistance profiles against 25 clinically-relevant antimicrobials were determined by broth microdilution; resistant phenotypes were observed for at least 15 of the 25 antibiotics tested in all isolates except one. Specifically, 8/10 isolates were carbapenem-resistant and 7/10 isolates were colistin-resistant. The *bla*_NDM-1_ and *bla*_OXA-48_ carbapenemase genes were present in 7/10 and 5/10 isolates, respectively; including 2 isolates carrying both genes. No plasmid-mediated determinants for colistin resistance (e.g. *mcr*) were detected, but disruptions and mutations in chromosomal loci (i.e. *mgrB* and *pmrB*) previously reported to confer colistin resistance were observed. A *bla*_OXA-48_-carrying IncL/M-type plasmid was found in all *bla*_OXA-48_-positive isolates. The application of WGS to molecular epidemiology and surveillance studies, as exemplified here, will provide both a more complete understanding of the global distribution of MDR isolates and a robust surveillance tool useful for detecting emerging threats to public health.

## Introduction

The Gram-negative bacterium *Klebsiella pneumoniae* is a clinically relevant pathogen that has a propensity to acquire multidrug resistance (MDR), thus limiting therapeutic options for treating community-acquired and nosocomial infections such as pneumonia, septicemia, wound, and urinary tract infections (UTIs) [1]. MDR and extensively drug-resistant (XDR) strains are defined as non-susceptible to at least one agent in three or more antimicrobial classes or non-susceptible to at least one agent in all but two or fewer antimicrobial classes, respectively [2]. The rapid worldwide spread of MDR *K*. *pneumoniae*, as well as other *Enterobacteriaceae*, poses a serious threat to global health [3]. *K*. *pneumoniae*, the most common *Klebsiella* species causing human infections, is one of the top three pathogens of international concern documented in the 2014 World Health Organization (WHO) Global Report on Surveillance of Antimicrobial Resistance [4].

Extended-spectrum β-lactamases (ESBLs) are bacterial enzymes that hydrolyze and inactivate most β-lactam antibiotics such as penicillins, broad-spectrum cephalosporins, and monobactams, but not cephamycins or carbapenems. Their production by bacterial pathogens confers resistance against a number of commonly used classes of β-lactam antibiotics and primarily restricts the choice of antimicrobial therapy to carbapenem antibiotics [5]. Thus, carbapenems are often employed as last resort antibiotics for the treatment of severe infections caused by MDR bacterial pathogens [1]. The acquisition of carbapenemases by *Enterobacteriaceae* has thus been especially worrisome as it threatens the clinical utility of these important therapeutic agents [1]. Indeed, the United States Centers for Disease Control and Prevention (CDC) has identified carbapenem-resistant *Enterobacteriaceae* (CRE) as one of the most urgent MDR threats [6].

The most common carbapenemases identified in *K*. *pneumoniae* to date are: i) class A β-lactamases (e.g., *K*. *pneumoniae* carbapenemase; KPC); ii) class B β-lactamases/metallo- β-lactamases (e.g., New Delhi metallo-β-lactamase-1 [NDM-1]), and iii) class D β-lactamases (e.g., oxacillinase-48; OXA-48-like carbapenemases) [1]. While these plasmid-encoded carbapenemases have been increasingly reported worldwide, their prevalence varies geographically [1,3].

Therapeutic options to treat infections caused by MDR carbapenemase-producing *K*. *pneumoniae* strains are limited to drugs that are less effective, more toxic, and/or not widely available, such as colistin (polymyxin E), polymyxin B, fosfomycin, tigecycline, and select aminoglycosides [1]. In the last few years, resistance to colistin has emerged due, in part, to its extensive use in livestock feed [7]. Endogenous resistance to colistin in *K*. *pneumoniae* can result from any of several genetic changes, including alteration of the *mgrB* gene and several non-synonymous point mutations in the genes encoding the two-component regulatory systems PhoPQ and PmrAB [8–10].

The continued emergence of antibiotic resistance in *K*. *pneumoniae*, and more broadly *Enterobacteriaceae*, presents a considerable clinical challenge. The steep decline in the discovery of effective antibiotics by pharmaceutical companies further exacerbates the threat posed by MDR pathogens. Rapid, accurate detection and characterization of antimicrobial resistance determinants and genomic mutations conferring resistance are crucial to countering the mounting burden of infections caused by MDR bacteria. Such information could help direct hospital resources to prevent nosocomial spread of MDR organisms and guide best-choice antimicrobial therapy to improve patient outcomes [11]. Unfortunately, some phenotypic detection methods can be unreliable for the detection of carbapenemase-producing bacteria, depending on the test used and the carbapenemase produced. For instance, the Modified Hodge Test (MHT) is a simple and easy laboratory test based on the inactivation of a carbapenem by a carbapenemase-producing isolate. While this culture-based test, originally the only Clinical Laboratory Standards Institute (CLSI)-recommended carbapenemase screening method [12], performs well for the detection of KPC and OXA-48 producers, it often fails to detect NDM-producing organisms [13]. Since 2017, CLSI has recommended two additional phenotypic tests (Carba NP, and mCIM) and removed MHT early 2018 [14].

Hospital laboratories are increasingly using whole genome sequencing (WGS) for the unambiguous identification of previously identified and characterized genes encoding antimicrobial resistance determinants [11]. This method can be used to identify resistance genes located on both the bacterial chromosome and on mobile genetic elements, as well as to track the emergence and persistence of resistance in previously susceptible bacterial pathogens [11]. Additionally, WGS provides a wealth of data that can be used for multiple analyses (e.g., concurrently determining sequence types or the presence of both virulence and resistance genes), thus helping to optimize resources and support appropriate clinical intervention. It is imperative to track the spread of existing determinants of antimicrobial resistance to previously susceptible organisms and recognize the emergence of new or novel combinations of determinants [15].

In the current study, we have characterized the resistome of carbapenem- and colistin-resistant clinical isolates of *K*. *pneumoniae* that were isolated in Pakistan between 2010 and 2013. Our findings provide important insight into the genetic diversity of these challenging MDR bacterial pathogens in a high prevalence area of the world. The characterization of these historical isolates will help facilitate an understanding of the emergence and spread of antimicrobial resistance in *K*. *pneumoniae*. Further, WGS-based context will help inform hospital infection control measures and aid the elucidation of contributing factors that promote the development of antimicrobial resistance in the non-hospital environment. Such information provides an essential foundation to support the development of novel diagnostic and therapeutic strategies for detecting and treating infections caused by MDR bacteria.

## Material and methods

### Samples

**Fig 1:**
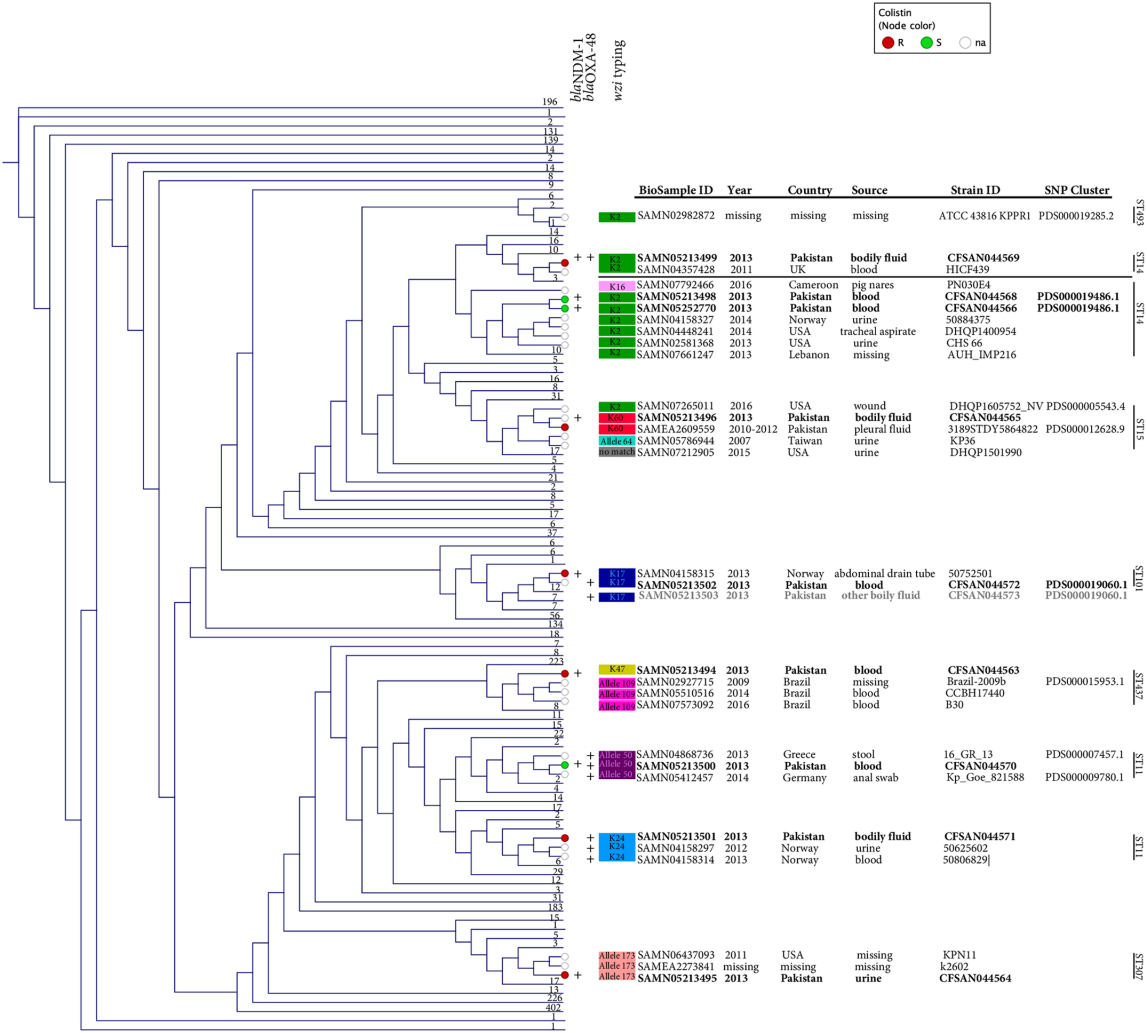
Cladogram of the Kmer distance tree derived from the NCBI Pathogen Detection database comprising 5169 *Klebsiella pneumoniae* isolates at time of writing. The tree also shows MLST, *wzi* typing, and the presence of *bla*_OXA-48_ and *bla*_NDM-1_ genes for the 10 clinical isolates and sequence control strain (ATCC 43816), as well as their closest relatives. For space optimization, all other isolates were collapsed into straight lines comprising different numbers (aka leaves). Colistin susceptibility is indicated with different colored dots: green (susceptible); red (resistant); and white (no information). The identification for the BioSample and SNP cluster (when available) is also provided. The complete tree file from NCBI Pathogen detection (PDG000000012.284.reference_target.tree.asn—as of March 24th, 2018), can be downloaded at https://doi.org/10.6084/m9.figshare.5708347.v2 and can be opened with NCBI software Genome Workbench (https://www.ncbi.nlm.nih.gov/tools/gbench/). Given that the kmer tree only includes a reference isolate from each SNP clusters plus singleton isolates, CFSAN044573 (marked in gray) is not included in the original tree but it is listed here for completeness of information.

Ten MDR clinical isolates of *K*. *pneumoniae* that were cultured from blood, urine, or other sites (e.g. wound) between 2010 and 2013 (Fig 1) were obtained from the Department of Pathology and Laboratory Medicine at the Aga Khan University Hospital (AKUH) in Karachi, Pakistan. Limited clinical information was available for all isolates. Initial species identification and antimicrobial susceptibility testing (AST) were performed at AKUH using Vitek 2 (bioMérieux, France). To get a snapshot of the resistome of MDR *K*. *pneumoniae* in Pakistan, isolates had been randomly selected among those resistant to at least one carbapenem. Of the 10 selected isolates resistant to at least one carbapenem, 7 were resistant to colistin while 3 were susceptible (Table 1). Three of the isolates (CFSAN044564, CFSAN044572, CFSAN044573) were from the same patient who had been hospitalized multiple times.

**Table 1:** Antibiotic susceptibility profiles of MDR *K*. *pneumoniae* isolates subjected to resistome analysis.

		ID CFSAN0									
List of antibiotics (n = 25 agents)		44563	44564	44565	44566	44568	44569	44570	44571	44572	44573
**Penicillins**	Ampicillin	R	R	R	R	R	R	R	R	R	R
**β-lactam/ β-lactamase inhibitor combinations**	Amoxicillin-clavulanic acid	R	R	R	R	R	R	R	R	R	R
	Ampicillin-sulbactam	R	R	R	R	R	R	R	R	R	R
	Piperacillin-tazobactam	R	R	R	R	R	R	R	R	R	R
**Cephalosporins**	Cefazolin—C1G^a^	R	R	R	R	R	R	R	R	R	R
	Cefoxitin—C2G^b^	R	R	R	R	R	R	R	R	I	I
	Cefotaxime -C3G^c^	R	R	R	R	R	R	R	R	R	R
	Ceftazidime—C3G	R	R	R	R	R	R	R	S	R	R
	Ceftriaxone—C3G	R	R	R	R	R	R	R	R	R	R
	Cefepime—C4G^d^	R	R	R	R	R	R	R	I	R	R
**Monobactams**	Aztreonam	R	R	R	R	R	R	R	S	R	R
**Carbapenems**	Imipenem	R	R	R	R	R	R	R	R	I	I
	Doripenem	R	R	R	R	R	R	R	R	S	S
	Meropenem	R	R	R	R	R	R	R	R	S	S
	Ertapenem	R	R	R	R	R	R	R	R	R	R
**Aminoglycosides**	Amikacin	R	R	R	R	R	R	R	S	R	R
	Gentamicin	R	R	R	R	R	R	R	I	R	R
	Tobramycin	R	R	R	R	R	R	R	I	R	R
**Fluoroquinolones**	Ciprofloxacin	R	R	R	R	R	R	R	R	R	R
	Levofloxacin	R	I	R	S	S	S	R	R	R	R
**Folate Pathways inhibitors**	Trimethoprim-sulfamethoxazole	R	R	S	R	R	R	R	S	S	S
**Polymyxins**	Colistin	R	R	R	S	S	R	S	R	R	R
**Chloramphenicol**	Chloramphenicol	R	S	S	S	S	S	R	I	S	S
**Tetracyclines**	Tetracycline	R	S	S	R	R	S	S	S	S	S
	Tigecycline	S	S	S	S	S	S	S	S	S	S

β-lactam antibiotic class are shaded grey. Red/R indicates resistance, green/S susceptibility, and yellow/I intermediate. Strains from the same patient are underlined.

^**a**^C1G: first generation cephalosporin

^**b**^C2G: second generation cephalosporin

^**c**^C3G: third generation cephalosporin

^**d**^C4G: fourth generation cephalosporin

The 10 selected isolates were sent to University of Virginia (Charlottesville, VA, USA [UVA]) for further studies [16]. DNA was extracted using the Qiagen DNeasy Blood & Tissue Kit (Germantown, MD, USA). DNA samples prepared at UVA were sent to the Center for Food Safety and Applied Nutrition of the US Food and Drug Administration (College Park, MD, USA) where WGS was carried out. *K*. *pneumoniae* ATCC 43816 (CFSAN044574) was included as a sequencing control. All 10 isolates were sent to the Centers for Disease Control and Prevention (Atlanta, GA, USA) for confirmation of AST testing using the reference BMD method.

### Antimicrobial susceptibility testing (AST)

The MIC values for 25 antimicrobial agents were determined by BMD, according to CLSI guidelines [12,14,17]. *Escherichia coli* ATCC 25922 and *Pseudomonas aeruginosa* ATCC 27853 were used as control strains for antimicrobial susceptibility testing. Susceptibilities were interpreted using clinical breakpoints established by the CLSI for 23/25 drugs. The European Committee for Antimicrobial Susceptibility Testing (EUCAST) breakpoints [18] were used for colistin and tigecycline, since CLSI breakpoints are not currently available. Table 1 indicates the antimicrobials agents tested, as well as isolates susceptibilities.

### Sequencing and assembly of genomes

DNA libraries and genomic assemblies were prepared as previously described [19]. Briefly, DNA was prepared using the Nextera XT DNA Library Preparation Kit (Illumina, San Diego, CA, USA), and WGS was carried out on a MiSeq platform using the 2 × 250 bp paired-end MiSeq Reagent Kit v2 (Illumina, San Diego, CA, USA). SPAdes Genome Assembler (version 3.9) was used to obtain *de novo* assemblies, which are available under accession numbers MAG(C/E/F/G/H/I/J/K/L/M)00000000 [19]. To support global distribution and provide epidemiological linkages, all genomes in this study were uploaded to the publicly available NCBI Pathogen Detection website (https://www.ncbi.nlm.nih.gov/pathogens/).

DNA sequences were analyzed using the IS finder web resource (https://www-is.biotoul.fr/blast.php), to identify insertion sequences (ISs) and IS fragments [20]. Potential integrons were further annotated using INTEGRALL (http://integrall.bio.ua.pt) [21]]. Identified amino acid substitutions were checked in the Protein Variation Effect Analyzer tool (PROVEAN—http://provean.jcvi.org/index.php) to predict their effect on the biological function of the protein (i.e. neutral or deleterious) [22]. Average nucleotide identities (ANIs) between genomes of interest were calculated using JSpeciesWS (http://jspecies.ribohost.com) [23]. Progressive Mauve was used to align genomes to identify conserved or disparate regions and/or SNPs (http://darlinglab.org/mauve/mauve.html) [24]. PHASTER (PHAge Search Tool Enhanced Release) was used for the identification and annotation of prophage sequences within obtained genomes (http://phaster.ca) [25].

### Molecular typing of isolates and plasmid profiling

The Bacterial Isolate Genome Sequence Database (BIGSdb) (http://bigsdb.web.pasteur.fr/perl/bigsdb/bigsdb.pl?db=pubmlst_klebsiella_seqdef_public) was used to characterize each of the *K*. *pneumonia*e isolates by determining the ST (multi-locus sequence type) and capsular serotype. In particular, to predict the capsular (K) types of the examined isolates (among the currently identified 79 K types), we used the sequences of the *wzi* gene, one of the six conserved genes in the *cps* locus [26]. PlasmidFinder (https://cge.cbs.dtu.dk/services/PlasmidFinder/) was used to type plasmids via the identification of major incompatibility (Inc) groups in *Enterobacteriaceae* species [27]. The minimum percentage of sequence identity was set at 100%, with an alignment length of >98%.

To increase the size of our analysis, we have included comparisons of the 10 isolates sequenced herein with more than 5100 other *K*. *pneumoniae* isolates obtained worldwide, using the NCBI Pathogen Detection tool (https://www.ncbi.nlm.nih.gov/pathogens/). This pipeline uses WGS data to: i) produce a phylogenetic tree based on Kmer distance; ii) perform single-linkage clustering (with a SNP distance of 50 SNPs) to find closely related isolates, and iii) determine the antibiotic resistance (AMR) profiles of isolates. A Kmer distance tree was obtained from NCBI Pathogen Detection on March 24^th^, 2018. This Kmer tree includes a reference isolate from each SNP clusters plus singleton isolates, which are not currently included in SNP clusters.

### Identification of resistance determinants

Three different approaches were used to identify antimicrobial resistance genes and select efflux pumps: ResFinder, the Comprehensive Antibiotic Research Database (CARD), and NCBI Pathogen Detection. Assembled genomes were uploaded to the web resource ResFinder v2.1 (http://cge.cbs.dtu.dk/services/ResFinder/) for the identification of acquired antimicrobial resistance genes [28]. The minimum percentage of sequence identity threshold was set at 98%, with an alignment length of at least 80%. Assemblies were also analyzed with the tools available at CARD for strict and perfect hits, with identity >96% (http://arpcard.mcmaster.ca) [29], and using the NCBI Pathogen Detection tool. When conflicting nomenclatures were encountered, the NCBI nomenclature was used. The presence of acquired colistin resistance genes (i.e. *mcr* variants) was determined using the database resources described above. Disruptions or alterations of chromosomal loci conferring colistin-resistance (e.g. *mgrB*, *pmrAB*, *phoPQ*) were determined *in silico* using CLC Genomics Workbench v.9 (CLC Bio, Aarhus, Denmark) [8–10]. Chromosomal point mutations in the Quinolone Resistance Determining Region (QRDR) of *gyrA*, *gyrB* and *parC*, *parE* genes were investigated for characterization of quinolone resistance using PointFinder [30].

## Results

### Antimicrobial susceptibility profiles

Isolate-specific antimicrobial susceptibilities are shown in Table 1. One of the *bla*_NDM_-producing isolates, CFSAN044563, was resistant to 24/25 antibiotics tested, and susceptible to only tigecycline. Conversely, one of the *bla*_OXA-48_-producing strains, CFSAN44571, exhibited the highest number of intermediate and susceptible results (n = 10 antibiotics). Interestingly, CFSAN044572 and CFSAN044573 were susceptible to meropenem and doripenem but intermediate to imipenem.

All isolates were resistant to the same 9 antibiotics (8 β-lactams and ciprofloxacin). Non-susceptibility to 3 other β-lactam antibiotics was observed in 70% of the isolates, including two isolates (CFSAN044572 and CFSAN044573) which were intermediate to cefoxitin and imipenem and one strain (CFSAN044571) intermediate to cefepime (Table 1). For carbapenems, 80% of the strains were resistant to all four carbapenems tested. Of the remaining antibiotics, 90% of the strains were resistant to all aminoglycosides; 70% to colistin; 60% to trimethoprim-sulfamethoxazole; 30% to tetracycline and 40% to chloramphenicol (Table 1). All isolates were susceptible to the tetracycline derivative tigecycline.

### Molecular typing and plasmid profiling

The 10 *K*. *pneumoniae* clinical isolates analyzed here were determined to belong to 5 different sequence types (STs): ST11 (n = 3 isolates), ST14 (n = 3), ST15 (n = 1), ST101 (n = 2), and ST307 (n = 1) (Fig 1 and Table 2). CFSAN044574 (ATCC 43816) belonged to ST493, as previously reported in the Bacterial Isolate Genome Sequence Database (BIGSdb). Examination of the *wzi* sequences identified 7 alleles, which were associated with specific capsular types (Fig 1). The most common capsular type was K2 (n = 3 isolates); followed by K17 (n = 2); K24, K47, and K60 (n = 1 each). The K2 capsular type was present in multiple STs (ST14, and ST15). Two isolates possessed *wzi* alleles that, based on BIGSdb, were either associated with multiple capsular serotypes (allele 50, CFSAN044570) or not yet assigned to a specific capsular serotype (allele 173, CFSAN044564) (Fig 1).

**Table 2:** List of antimicrobial resistance genes and plasmid replicon-types.

		ID CFSAN0									
		ST11			ST307	ST14			ST15	ST101	
**Class**	**Gene**	**44570**	**44563**	**44571**	**44564**	**44569**	**44566**	**44568**	**44565**	**44572**	**44573**
A	*armA*		**x**		**x**	**x**	**x**	**x**			
	*rmt*	**F1**							**C**	**F1**	**F1**
	*aadA*	2	1				1	1			
	*aac(3)-*	IIa	IIa	II	IIa		IIa	IIa		IIa	IIa
	*aph(3')-*	**Ia**	VI								
	*aph(3'')-Ib*		x		**x**	**x**	**x**	**x**		**x**	**x**
	*aph(6)-Id*		**x**		**x**	**x**	**x**	**x**			
	*aac(6')-Ib*								x	x^a^	x
	*aac(6')-Ib-cr*	x	x	x	x	x	x	x			
B	*bla*_CTX-M-15_	**x**	**x**		**x**	**x**	**x**	**x**	**x**	**x**	**x**
	*bla*_SHV-_	**11**	**11**	**11**	**28**	**28**	**28**	**28**	**28**	**1**	**1**
	*bla*_OXA-1_	**x**	**x**		**x**	**x**	**x**	**x**		**x**	**x**
	*bla*_OXA-10_		**x**				**x**	**x**			
	*bla*_OXA-48_	**x**		**x**		**x**				**x**	**x**
	*bla*_NDM-1_	**x**	**x**		**x**	**x**	**x**	**x**	**x**		
	*bla*_TEM-1_		**x**		**x**		**x**	**x**		**x**	**x**
	*bla*_CMY-_						**16**	**16**	**6**		
C	*cmlA5*		x				x	x			
	*catA1*		x								
F	*qnr*		**S1**			B1	B1	B1			
G	*ble*	**x**	**x**		**x**	**x**	**x**	**x**	**x**		
M	*mph*	**A**			**E**	**E**	**E**	**E**			
	*Mrx*	**x**									
R	*arr*	**x**	**x**				**x**	**x**		**x**	**x**
S	*sul*	**1**	1	1	**1,2**	**1**	**1,****2**	**1,****2**	**1**	**2**	**2**
Te	*tet(A)*		x				**x**	**x**			
Tr	*dfrA*	**12**	**1**		14	14	14	14			
Efflux pumps	*qacEdelta1*	**x**	**x**	**x**	**x**	**x**	**x**	**x**	**x**		
	*msr(E)*		x		x	x	x	x			
Plasmid replicon types	IncL/M	x		x		x				x	x
	IncFIB(pQiL)		x	x	x	x					
	IncFIB(Mar)		x								
	IncFIB(K)	x									
	IncFII(pKPX1)	x								x	x
	IncFII(K)	x									
	IncHI1B		x								
	IncA/C2			x			x	x	x		

Classes of antimicrobial resistance genes are indicated as follows: A (Aminoglycosides); B (β-lactams); C (Chloramphenicol); F (Fluoroquinolones); G (Glycopeptides); M (Macrolide); R (Rifampicin); S (Sulfonamides); Te (Tetracycline); Tr (Trimethoprim). An x or an allele designation indicates the detection of the gene using NCBI Pathogen Detection, ResFinder (98%ID threshold, 80% minimum length), and/or CARD (perfect and strict hits, with identity >96%). A bolded x or allele designation indicates 100% identity. Possible gene duplications are underlined. For the plasmid replicon analysis, an x indicates the presence of a replicon type as determined using by PlasmidFinder (%ID threshold: 100% and a query vs. HSP length ratio of >98%). The *oqxAB* genes, which were present in all isolates, are not listed in the Table. In CFSAN044572, *aac(6’)-lb* is annotated as a partial sequence.

None of the 10 strains was grouped in a SNP cluster with any other *K*. *pneumoniae* strains in the NCBI Pathogen Detection database, containing 5169 strains at time of writing. However, 2 pairs of strains (CFSAN044572/CFSAN044573 and CFSAN044566/CFSAN044568) were found to be closely related to each other and grouped in a SNP cluster. Specifically, CFSAN044572 and CFSAN044573 were grouped in SNP cluster PDS000019060.1, with a calculated distance between them of an average of 800 SNPs. CFSAN044566 and CFSAN044568 were grouped in SNP cluster PDS000019486.1, with a calculated distance of 1 SNP. Additionally, CFSAN044574 (ATCC 43816) was grouped in SNP cluster PDS000019285.1 with KPPR1, a spontaneous rifampin-resistant isolate derived from ATCC 43816 [31], confirming the accuracy of sequencing and assembly (Fig 1). The list of isolates, their AMR genotypes, overall results, and Kmer tree are available at the *K*. *pneumoniae* species webpage at NCBI Pathogen Detection (https://www.ncbi.nlm.nih.gov/pathogens/). The isolates reported here represent a diverse population, based on the spectrum of observed STs and differences in resistomes. In all cases, K types were clearly defined based on either the ST or cluster, except for ST15 and a subset of ST11 in which four and two different capsular types were observed, respectively (Fig 1). Among the ST11 isolates, CFSAN044570 (ST11, 2010, positive for both *bla*_NDM-1_ and *bla*_OXA-48_) clustered with a ST11 strains isolated in 2013 from stool in Greece (16_GR_13) and two other ST11 isolates linked to a *bla*_OXA-48_-positive *K*. *pneumoniae* outbreak in Germany in 2013–2014 (SNP cluster PDS000009780.1, n = 2). These latter two isolates were obtained from skin and rectal swabs and each was associated with colonization in refugees from North Africa (Fig 1). CFSAN044571 clustered with two ST11 strains isolated in Norway: 50625602 from urine (2012) and 50806829 from blood (2013) (Fig 1). CFSAN044563 grouped with *bla*_KPC-2_ positive isolates from Brazil belonging to ST437 (SLV—single locus variant of ST11): Brazil-2009b (belonging to SNP cluster PDS000015953.1, n = 4); CCBH17440 (2014, sepsis/blood); and B30 (2016, blood). Of the three ST14 isolates, the genomes of CFSAN044566 and CFSAN044568 were most closely related as they were grouped in a SNP cluster, and differed by 1 SNP. CFSAN044569 grouped with HICF439, a ST14 strain from the UK (2011, blood/bacteremia). The ST15 isolate CFSAN044565 grouped with other ST15 strains: DHQP1501990 (2015, urine, USA); DHQP1605752_NV (2016, wound, USA, SNP cluster PDS000005543.4, n = 5); KP36 (2007, urine, Taiwan, 2007) [32]; and 3189STDY5864822. DHQP1605752_NV has been recently described as one of the first *K*. *pneumoniae* isolates in the US to be non-susceptible to all 26 drugs tested, including all ß -lactams, colistin, and tigecycline. The isolate carried two plasmids (IncA/C2 and IncFIB), and four ß-lactamase genes (plasmid-mediated *bla*_NDM-1_ and *bla*_CMY-6_, and chromosomal *bla*_CTX-M-15_ and *bla*_SHV-28_) [33]. 3189STDY5864822 belongs to SNP cluster PDS000012628.9, comprising 40 clinical isolates from Pakistan, collected between 2010 and 2012, and of which 4 were *bla*_NDM-1_ positive. The two ST101 isolates were highly related, sharing an average ANI of 99.9% and belonged to the same SNP cluster, as described above. This observation is consistent with their isolation from different sites of the same patient, blood for CFSAN044572 and catheter tip for CFSAN044573. The same patient was also infected with another strain (CFSAN044564) that belonged to a different ST (ST307), which is located on a separate branch on the phylogenetic tree (Fig 1). The closest relatives for this isolate were two ST307 clinical isolates: KPN11 from the US (2011), and k2602 from the BSAC Resistance Surveillance program (Fig 1).

Eight plasmid replicon-types were observed with a percent ID of 100%: IncL/M(pOXA-48) (n = 5 isolates), IncA/C2 and IncFIB(pQil) (n = 4); IncFII(pKPX1) (n = 3); and IncFIB(Mar), IncHI1B, IncFII(K) and IncFIB(K) (n = 1 each) (Table 2). The IncL/M(pOXA-48) replicon was present in the five *K*. *pneumoniae* strains in which the *bla*_OXA-48_ gene was detected (Table 2). No clear relationship between replicon type and ST was observed, except for the two ST101 isolates that shared the same plasmid profile (Table 2). No replicon-types were detected in the control strain *K*. *pneumoniae* ATCC 43816 (CFSAN044574), since that strain does not contain any plasmid [30]. Overall, the highest number of different replicon-types was observed in ST11 strains (n = 8 types), followed by ST14 (n = 3), ST101 (n = 2), and ST307 and ST15 (n = 1 each) (Table 2).

### Antimicrobial-resistance determinants

In most cases, ResFinder, CARD and NCBI Pathogen Detection were in agreement in identifying the predominant antibiotic resistance genes; however, some differences in nomenclature and reference sequences were noted. Table 2 shows a selection of the antimicrobial resistance determinants that were identified using the three platforms (n = 41 genes). Overall, ResFinder and the NCBI pipeline detected, on average, a smaller number of genes (19 per strain) than CARD (78 per strain). This discrepancy is due to the former two primarily identifying plasmid-associated resistance determinants and not chromosomal loci associated with antibiotic resistance.

According to all three platforms, the highest diversity of resistance genes was observed for aminoglycosides (n = 13 genes) and β-lactams (n = 11), with at least two resistance determinants present for each antibiotic class present in every isolate (Table 2). Consistent with BMD results, CFSAN044563 had the highest number of resistance determinants (n = 23 genes), while CFSAN044571 had the fewest (n = 6); both were identified as ST11 isolates. Among the genes present in multiple isolates, 4 were associated with a specific ST: *bla*_SHV-1_ in ST101; *bla*_SHV-11_ in ST11; *qnrB1* and *bla*_CMY-16_ in ST14. The remaining genes were found in isolates belonging to different STs (Table 2).

Two ESBL genes were identified (*bla*_CTX-M-15_ and *bla*_SHV-28_). *bla*_CTX-M-15_ was the most frequent ESBL gene detected (n = 9 isolates) and was present in combination with *bla*_SHV-28_ (n = 5) or the following non-ESBL genes: *bla*_TEM-1_ (n = 6) and other *bla*_SHV_ genes (n = 5). No *bla*_KPC_ genes were found. *bla*_NDM-1_ was the most frequently detected carbapenemase gene (n = 7 isolates), followed by *bla*_OXA-48_ (n = 5). In two isolates (CFSAN044570 and CFSAN044569), both *bla*_NDM-1_ and *bla*_OXA-48_ were present (Table 2). CFSAN044571 possessed the fewest β-lactam and carbapenem resistance genes, and was susceptible to ceftazidime (C3G) and aztreonam (monobactam), but had intermediate susceptibility to cefepime (C4G).

None of the ten strains analyzed possessed any of *mcr* genes, the plasmid-borne determinants of colistin resistance [34]. In all cases, chromosomal mutations associated with colistin resistance were identified. Specifically, while no amino acid substitutions were observed in *mgrB*; disruption of *mgrB* and point mutations within *prmB* were observed in colistin-resistant strains (Table 3). The *mgrB* of strains CFSAN044564, CFSAN044565, and CFSAN044569 was disrupted by three different classes of IS families (ISKpn25, IS5, and IS1) inserted at different nucleotide positions (*mgrB23*, *mgrB38*, and *mgrB45*, respectively) (Table 3). In one isolate (CFSAN044563), no *mgrB* gene sequence could be identified, indicating the possible loss of the entire *mgrB* locus. No substitutions were observed in *phoP*, while only changes encoding neutral amino acid substitutions were found in *pmrA* and *phoQ* (S1 Table). In contrast, multiple non-synonymous substitutions were observed in *pmrB* (n = 14 substitutions), with most of them (n = 10) being considered deleterious to protein structure/function. A mutation resulting in a Thr157Pro amino acid substitution, previously confirmed to be responsible for colistin resistance [9], was found in all (n = 3) of the colistin-resistant strains with an intact *mgrB* gene (Table 3).

**Table 3:** Disruptions/insertion in the *mgrB* gene and point mutations causing neutral and deleterious amino acid substitutions (in pink) in the *pmrB* gene.

	*mgrB*	*pmrB*													
ID CFSAN0		T93P	N110T	T112P	T127P	T128P	L130P	L141P	V151G	T157P	L159P	L164P	L213M	A246T	R256G
44563	deletion	x		x	x	x									
44564	ISKpn25 at *mgrB23*												x		
44565	IS5-like at *mgrB38*		x	x		x					x			x	x
44569	IS1-like at *mgrB45*													x	x
44571	intact									x					
44572	intact									x					x
44573	intact						x	x	x	x	x	x			x
44570	intact														
44566	intact							x						x	x
44568	intact													x	x

Colistin-resistant strains are underlined, and amino acid substitutions known to be associated with colistin resistance are in bold. The genome sequence for *K*. *pneumoniae* HS11286 was used as a reference (GenBank assembly accession GCA_000240185.2).

For quinolone-resistant isolates, three different genotype profiles were observed: i) a *gyrA* mutation (S83I) and a *parC* mutation (S80I) in 3 isolates (CFSAN044563, CFSAN044564 and CFSAN044570); ii) 2 *gyrA* mutations (S83F/Y) and a *parC* mutation (S80I) in 4 isolates (CFSAN044565, CFSAN044571, CFSAN044572, and CFSAN044573); and no mutations at all in 3 isolates (CFSAN044566, CFSAN044568, and CFSAN044569). The following plasmid-mediated quinolone resistance genes were identified: *aac(6’)-lb-cr* (6 isolates), *qnrB1* (all ST14 isolates), and *qnrS1* (1 ST11 isolate), along with the *oqxAB* efflux pump (all isolates). The 3 isolates (CFSAN044566, CFSAN044568, and CFSAN044569) with no mutations in the topoisomerase type II enzymes, both carried *qnrB1* and *aac6’-Ib-cr*. Additional genes detected included the *tet(A)* gene in the three tetracycline-resistant strains; and *dfrA*/*sul1* in the 6 trimethoprim-sulfamethoxazole resistant-strains (Table 2).

### *bla*_NDM-1_ common region and gene duplication

**Fig 2:**
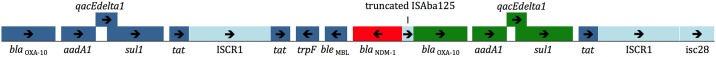
Schematic representation of the genetic environment of the *bla*_NDM-1_ common region and gene duplication organization in ST14 isolates CFSAN044566 and CFSAN044568. *bla*_NDM-1_ is in red, other genes in blue, duplicated copies in green, and insertion sequences are in light blue. The *bla*_NDM-1_ common region includes a truncated ISaba125, the *bla*_NDM-1_ gene, the bleomycin resistance protein (*ble*_MBL_) and phosphoribosylanthranilate isomerase (*trpF*) genes. Duplicate copies of four genes (*bla*_OXA-10_, *aadA1*, *qacEdelta1*, and *sul1*) were found. The *tat* gene is present in two copies split in the middle in one instance. A straight line indicates gaps between the ORFs.

In the seven *bla*_NDM-1_-positive isolates, the regions immediately flanking the *bla*_NDM-1_ locus were conserved and included the bleomycin resistance protein (*ble*_MBL_) and the N-(5’-phosphoribosyl)anthranilate isomerase (*trpF)* genes (Fig 2). In four isolates, the interrupted sequence of the left end of ISAba125 was located upstream of the *bla*_NDM-1_ gene, while the complete sequence of ISAba125 was present only in one isolate (CFSAN044563). In two of the isolates (CFSAN044566 and CFSAN044568), the original ISAba125-bracketed *bla*_NDM-1_ region seems to have been further mobilized by an ISEC28-mediated event downstream of a class 1 integron, thereby generating an extended multidrug-resistance scaffold and a duplication event (Fig 2).

Two copies of a gene cassette comprising *bla*_OXA-10_, *aadA1*, *qacEdelta1*, and *sul1* were present in CFSAN044566 and CFSAN044568 (Fig 2). The latter three genes were identical in both repeats and in both isolates. In the assembled genome of CFSAN044568, the first repeat of *bla*_OXA-10_ shows a non-synonymous mutation (G663A) that results in a premature stop codon. However, when reads were mapped back to the assembly, this mutation was not observed, possibly due to an assembly error caused by repetitive nucleotide sequences in this region. In both isolates, the insertion of a partial ISAba125 element truncates the first 33 nucleotides of the second copy of *bla*_OXA-10_.

To evaluate the number of reads specific for the genes seen in duplicate copies and therefore estimate the sequence coverage in CFSAN044566 and CFSAN044568, CLC Genomics Workbench v.9 was used to map raw sequence reads to reference sequences for the *bla*_NDM-1_, *bla*_OXA-10_, *aadA1*, *qacEdelta1* and *sul1* genes (NG_049326.1, NG_050979.1, NG_052030.1, NG_048042.1, NG_048100.1, respectively). CFSAN044563 was used as a control as all 5 genes were present without duplication. We observed a higher average coverage for *bla*_OXA-10_, *aadA1*, *qacEdelta1*, and *sul1* in CFSAN044566 (80X, 80X, 85X, 81X, respectively) and CFSAN044568 (127X, 114X, 117X, 94X) compared to *bla*_NDM-1_ (40X and 58X). In CFSAN044563, the coverages were the following: *bla*_OXA-10_ (39X), *aadA1* (43X), *qacEdelta1* (58X) and *sul1* (46X), and *bla*_NDM-1_ (37X).

## Discussion

WGS data for 10 MDR *K*. *pneumoniae* strains isolated between 2010 and 2013 in Pakistan were analyzed to provide a comparative genetic context for carbapenem and colistin resistance that will help inform infectious diseases epidemiology and the identification of antimicrobial resistance determinants. Knowledge of the genomic content of these historical isolates will also be useful for elucidating the spread of antimicrobial resistance in *K*. *pneumoniae*. The analyzed isolates represent a diverse population as indicated by the Kmer distance tree, assigned ST and capsular types, and the resistomes.

WGS findings reflect the considerable antimicrobial resistance displayed by these isolates to multiple antibiotics, with resistance to β-lactams (including carbapenems) and aminoglycosides, fluoroquinolones, and colistin ranging from 60% to 100%. Only 30–40% of the isolates exhibited resistance to tetracycline and non-susceptibility to chloramphenicol, which may reflect the reduced clinical use of these agents in Pakistan [35]. Indeed, a previous study reported that cephalosporins were the first option for empirical treatment of bacterial infections caused by MDR-pathogens in Pakistan and that there was limited use of carbapenems due to their high cost [36]. However, sales of carbapenems almost tripled between 2005 and 2010 [35], possibly in response to a rise in infections caused by ESBL-producing pathogens.

The isolates examined in this study belong to either established (ST11, ST15, and ST14) or emerging (ST101 and ST307) antibiotic resistant high-risk clones of *K*. *pneumoniae* [5]. *K*. *pneumoniae* ST258 emerged in the middle 2000s in the US and has become a worldwide-propagated clone, along with its related variants belonging to clonal group 258 (CG258) [5]. ST11, a SLV of ST258, can capture multiple plasmids and is typically associated with MDR pathogens [5]. This ability is reflected by the different antimicrobial resistance and plasmid profiles of the 3 ST11 isolates we examined. Specifically, the three ST11 strains were highly diverse as they were represented by a NDM-1-producer (CFSAN044563), an OXA-48-producer (CFSAN044571), and one carrying both the *bla*_NDM-1_/*bla*_OXA-48_ genes (CFSAN044570). These 3 ST11 strains were either colistin-susceptible (CFSAN044570) or colistin-resistant through different chromosomal mechanisms of resistance (*mgrB* deletion or T157P mutation in *pmrB* for CFSAN044563 and CFSAN044571, respectively). The plasmid replicon-type and AMR profiles also differed within ST11, comprising between 3 and 4 different plasmid replicon-types (IncFIB, IncFII, IncL/M and IncA/C2) and sharing only 3 common AMR markers: *aac(3)-IIa*, SHV-11, and *qacEdelta1*. Non-susceptibility to chloramphenicol was observed in only two ST11 strains; one strain (CFSAN044563) which harbored both *cmlA5* and *catA1*; and one (CFSAN044570) which did not carry any genes associated with phenicol-resistance, thus suggesting the involvement of another mechanism (e.g.: overexpression of efflux systems). After alignment with MAUVE, a large region (contig MAGJ05, 110951 bp) was observed to be present in CFSAN044563 but absent in both CFSAN044570 and CFSAN044571. This contig contains a replicon with 95.73% identity to IncFIB from pKPHS1, but carries no resistance genes and is recognized by PHASTER as an intact phage, with a length of 110821 bp, *attL* and *attR* attachment sites, 117 proteins and a GC content of 48.92%. The most common related phage is listed as SSU5 from *Salmonella* [37], which has been described as a temperate phage with a circular plasmid prophage, as it is homologous to circular plasmids in several *Enterobacteriaceae* genomes [38]. Blasting of the MAGJ05 sequence showed different percentages of identity and query coverage vs.: SSU5 (83% identity with 48% coverage); and plasmids pKPHS1 (98% identity with 88% coverage), and pPMK1-B and p1605752FIB_2 (both 99% identity and 91% coverage). Additionally, the DEAD/DEAH box helicase gene (BAY54_20090 and BAY54_20675) in the MAGJ05 contig overlaps for 127 bp. Future research with long read sequence will be needed to confirm the genomic location and characteristics of this region.

Both ST14 and its SLV ST15 frequently carry and disseminate resistance determinants, such as multiple β-lactamases [1,5]. ST14 strains have been associated with pediatric and neonatal infections, sometimes carrying *bla*_NDM-1_ or *bla*_OXA-48_ [39–42], and ST15 strains have been reported in intensive care facilities [43]. Of the three ST14 strains, two bla_NDM-1_- possessing strains (CFSAN044566 and CFSAN044568), were susceptible to colistin and are closely related as they belong to the same SNP cluster and share a similar plasmid profile (IncA/C2), comprising 24 different AMR markers for 6 different antibiotic families: β-lactams (*bla*_CTX-M-15_, *bla*_SHV-28_, *bla*_OXA-1_, *bla*_OXA-10_, *bla*_TEM-1_, *bla*_CMY-16_); aminoglycosides (*armA*, *aadA1*, *aac(3)-IIa*, *aph3”-Ib*, *aph6-Id*, *aac6’-Ib*); chloramphenicol (*cmlA5*); cotrimoxazole (*sul1&sul2*), trimethoprim (*dfrA14*); macrolides (*msrE*); along with class 1 integron markers (*qacEdelta1*, *sul1*). The third ST14 strain (CFSAN044569), co-possessing *bla*_NDM-1_ and *bla*_OXA-48_, was colistin-resistant due to an IS1-like insertion at *mgrB45*, with a different plasmid profile and shared 14 out of the 24 detected AMR genes. The absence of the *tet(A)* gene in CFSAN044569 and the presence of alterations in either *mgrB* or *pmrB* in CFSAN044566/68 likely explains differences in susceptibility to tetracycline and colistin, respectively.

Beginning in 2014, ST307 has emerged in Italy replacing the predominant hyper-epidemic ST258 clone of *K*. *pneumoniae* [44]. A 2017 US study showed that, between 2011 and 2015, strains of CG307 were more prevalent than those belonging to CG258 [45]. ST307 has also been reported to be a major *bla*_CTX-M_ producing clone in Pakistan (2009–2010) [46], the US (2010) [47], Morocco (2012) [48], Serbia (2013–2016) [49], South Korea (2015) [50], and Colombia (2012–2014) [42], where it has been associated with infections with a mortality rate >50%. ST307 strains have been associated with capsular type *wzi*-173 [44], as also observed here.

ST101 has been described as an emerging pandemic clone found in several countries e.g. Romania, 2012 [51]; Japan, 2012 [52]; Spain, 2012–2014 [53]; Algeria, 2014–2015 [54]; and others [1]. The two ST101 strains from the same patient (blood and catheter) were closely related and showed a similar plasmid profile, except for the *aac6’-lb* gene which was not identified in the assembly for CFSAN044572. However, the *aac6’-lb* gene was annotated as incomplete and when raw reads from CFSAN044572 were mapped to the *aac6’-Ib* sequence from CFSAN044573, the full gene was observed. The Mauve alignment showed 1194 SNPs in 543 regions (annotated genes, hypothetical proteins and unannotated/intergenic regions), with n = 505 showing between 1–5 SNPs; 32 between 6–10 SNPs, and 6 with >10 SNPs. In particular, the highest numbers of SNPs were observed in a glutamate dehydrogenase (n = 17, BAY53_19965); followed by 2 unannotated regions (n = 13 and 12, respectively); and 3 genes with 11 SNPs each (a multidrug RND transporter, BAY53_01340; the competence/damage-inducible protein A, BAY53_20960; and the exodeoxyribonuclease V subunit alpha A, BAY53_6365). Additionally, their phage profile appears to be identical, with one incomplete, two questionable, and two complete phages identified by PHASTER (S2 Table). The two ST101 strains were likely resistant to colistin due to a T157P mutation in *pmrB*; however, there were 7 additional SNPs in *pmrB* between CFSAN044572 and CFSAN044573, 6 of which are non-synonymous: L130P, L141P, V151G, T157P, L159P, L164P. Three neutral substitutions (L213M, A246T, and R256G) have been previously observed, but they do not appear to be associated with colistin resistance. In fact, complementation with the wild-type *pmrB* gene in isolates with these substitutions did not restore colistin susceptibility. Additional reports also suggest that these *pmrB* mutations are not related to colistin resistance [55–57]. In particular, R256G has been observed in both colistin-resistant (13 out of 17) and colistin-susceptible (10 out of 20) isolates [58]. Overall, the 7 colistin resistant isolates had different chromosomal mutations associated with colistin resistance and belonged to 5 different sequence types, confirming that colistin resistance is multifactorial and chromosomal determinants are independent of the sequence types [59].

The isolates analyzed herein grouped largely based on their ST in the Kmer distance tree. Given the presence of different mobile elements, the Kmer distance tree could potentially be confounding for the tips of the tree; however, Kmer analysis is able to capture differences across genomes (e.g. mutations, insertions/deletions, recombination, and differences in gene content) [60]. WGS results are consistent with the average short-term evolutionary rate for the two ST101 isolates from the same patient, suggesting that diversity within individual patients is low. While CFSAN044572 and CFSAN044573 are indeed closely related, given the observed number of SNPs and considering the potential artifacts introduced by short reads technology and assembly, it is difficult to determine if some evolution events occurred within the patient or that the patient was concurrently infected by two closely related strains. Future research with long-read sequencing will be necessary to better elucidate the relationship between these two isolates. However, the isolation of a different strain (ST307) from the same patient clearly indicates a non-clonal relationship that may have resulted from a subsequent infection with a different strain or the presence of diverse pathogen populations within the same individual, each of which complicates empirical treatment.

Capsular polysaccharide (CPS) is one of the main virulence factors of *K*. *pneumoniae* and capsular types are related to the clinical severity of the infections [61]. *Wzi* encodes for an outer membrane protein involved in capsule attachment to the surface of the cell [26]. The identification of distinct ST15 lineages, as suggested by the different capsular types associated with this ST, has been linked to the circulation of distinct lineages with differences in relative occurrence, geographical, niche distribution, and/or host susceptibility [62]. A similar finding was observed herein for the capsular type related to *wzi*-109 of ST437 (Fig 1), a ST belonging to the same clonal complex of ST11 and frequently observed between 2007 and 2009 in Brazil [63].

The variety of AMR genes was higher among the *bla*_NDM-1_-positive isolates (up to 23 genes), than among the *bla*_OXA-48_-positive isolates (up to 11 genes). All 7 *bla*_NDM-1_-positive isolates identified herein are carrying both *bla*_CTX-M-15_, with either *bla*_SHV-28_ (n = 5) or *bla*_SHV-11_ (n = 2), along with additional β-lactamases (*bla*_OXA-1_, *bla*_OXA-10_ or *bla*_OXA-48_). The main AMR genes identified in most *bla*_NDM_-positive isolates of that study were *sul1* (n = 6), *armA*, *aph3”-Ib*, *aph(6)-Id* (n = 5), *mphE* (n = 4), *dfrA14* (n = 4), *qacEdelta1* (n = 7), and *msrE* (n = 5). All the *bla*_OXA-48_ positive isolates were resistant to colistin except one strain. Of the 5 *bla*_OXA-48_-positive isolates, 4 carried both *bla*_CTX-M-15_ and one isolate carried both *bla*_CTX-M-15_ and *bla*_SHV-28_. The only additional β-lactamase identified was *bla*_OXA-1_ (n = 4) for *bla*_OXA-48_-positive isolates. Other AMR determinants associated with *bla*_OXA-48_ were *rmtF1* (n = 3), *aac(3)-IIa* (n = 4), *aph3”-Ib*, *sul1* (n = 5), *qacEdelta1* (n = 3). The plasmid replicon-type IncL/M was identified in all *bla*_OXA-48_-positive isolates along with additional types: IncFIb (n = 1), IncFII (n = 2), IncFIB + IncFII (n = 1), IncFIB + IncA/C2 (n = 1).

Overall, clonal diversity was observed in *bla*_NDM-1_- and *bla*_OXA-48_-positive *K*. *pneumoniae* isolates, as they belonged to four and three different STs, respectively, and the *bla* genes were harbored on different plasmids, except for CFSAN044566 and CFSAN044568 which likely carry the same plasmid. This finding is in contrast to *bla*_KPC_-positive *K*. *pneumoniae*, which historically have been linked to ST258 and its related variant in CG258 [5]. Differences in geographical distribution likely explain the absence of *bla*_KPC_ genes; KPC *K*. *pneumoniae* isolates maybe be rare in Pakistan as no KPC-associated isolates/cases were reported in a 2015 literature review [64], and only 2 strains carrying *bla*_KPC-3_ were detected in a study analyzing Pakistani isolates obtained between 2012–2013 [65].

The concurrent presence of *bla*_NDM-1_ and *bla*_OXA-48_ has been described in isolates from Morocco, 2011 [66]; Tunisia, 2012 [67]; United Arab Emirates, 2011–2013 [68,69]; Turkey, Switzerland and Australia, 2013 [70–72]. Since then, these isolates have been commonly reported and include those with different combinations of *bla*_OXA-48_ variants with *bla*_NDM-_ genes [73–81]. In the NCBI Pathogen Detection database, the combination of any of the variants of *bla*_OXA-48_ [82] with any *bla*_NDM-_ gene [83], was observed in 34 out of 5278 isolates (0.64%) (as of April 18th, 2018). Two isolates were from the present study and the remaining were isolated between 2011–2017 in Vietnam, Europe, South Korea, Thailand and the US. The identification of both genes in two isolates of different STs, one from 2010 and one from 2013, appears to be the first description of *bla*_NDM-1_ and *bla*_OXA-48_ co-producing *K*. *pneumoniae* clones originating from Pakistan thus predating what is currently described in the literature.

The gene encoding OXA-48 β-lactamase was first identified in 2000; since then, this class D β-lactamase and its variants have become clinically significant worldwide [1]. As CRE-producing OXA-48-like enzymes may be difficult to recognize since they only weakly hydrolyze both cephalosporins and carbapenems, their incidence is possibly underestimated [3]. A highly-transferable pOXA-48 plasmid (IncL group), generally containing no other antibiotic resistance genes, was reported to be primarily responsible for spreading the *bla*_OXA-48_ gene in *K*. *pneumoniae* [1]. Confirming the ability of high-risk clones to accumulate resistance determinants, the *bla*_OXA-48_ gene was detected in strains belonging to different STs (ST101, ST11, and ST14). In particular, ST101 does not appear to have been previously observed in Pakistan. To the best of our knowledge, this is the first description of a *bla*_OXA-48_ producing *K*. *pneumoniae* strain of ST101 isolated in Pakistan. In fact, the only report of a *bla*_OXA-48_-producing *K*. *pneumoniae* linked to Pakistan appears to be from a December 2012 case of osteomyelitis in an infant that had sustained a burn injury in Karachi, Pakistan before returning to Canada, where the *bla*_OXA-48_ producing strain was isolated [84]. This highlights the complex issues and challenges presented by MDR organisms, as clinicians around the world need to be aware of global trends in antimicrobial resistance as focusing only on local patterns might not be sufficient to make prudent clinical decisions.

Seventy percent of the *K*. *pneumoniae* isolates examined were found to carry *bla*_NDM-1_, consistent with it being endemic in South Asia (i.e., Bangladesh, India, and Pakistan) [1]. Specifically, *bla*_NDM-1_ has been identified in the majority (75%) of carbapenem-resistant *K*. *pneumoniae* in 2011 in Pakistan [36]; since 2015, the country has been associated with single hospital outbreaks of NDM-producing isolates [64]. As observed for *bla*_OXA-48_, *bla*_NDM-1_ was associated with ST11, ST14, ST15, and ST307, which is troubling as these STs frequently contain *bla*_CTX-M_ and *bla*_KPC_, and represent both well-established and emerging clones. Additionally, our *bla*_NDM-1_-positive ST14 isolates belonged to serotype K2, which is considered to be one of the predominant virulent serotypes [61]. The *bla*_NDM_ genes have been found on a wide variety of different broad-host-range plasmids, thus facilitating the spread of such genes by horizontal gene transfer to various *Enterobacteriaceae* species [85]. Among the seven *bla*_NDM-1_ positive isolates, several different replicon-types associated with *bla*_NDM-1_ were identified, confirming the high variability of the *bla*_NDM-1_ genomic context.

The immediate genetic environment of the *bla*_NDM-1_ gene is conserved [85]. A *bla*_NDM-1_ common region usually comprises a truncated ISAba125 element immediately upstream of *bla*_NDM-1_, followed by the *ble*_MBL_ gene and the *trpF* gene (either complete or truncated) [85,86]. ISAba125 is thought to be responsible for the initial mobilization of *bla*_NDM-1_ from its progenitor [85,86]. In CFSAN044566 and CFSAN044568, the arrangement of the *bla*_NDM-1_ region suggests there were multiple insertion events, as two copies of an ISCR1 element are present. It may be that this region was first mobilized onto a plasmid by an ISAba125-mediated event, followed by transfer onto an IncA/C2-like plasmid by an ISCR1-mediated transpositional event. The presence of different IS elements has been suggested to aid the efficiency by which resistance genes spread and both ISCR1 and IS26 have been observed to facilitate the transposition and/or expression of resistance genes located near them [87]. The flanking genetic elements may act as hot spots for recombination, responsible for the mobilization of the conserved *bla*_NDM-1_ region and the high dissemination rate of *bla*_NDM-1_ worldwide, even without an apparent epidemiological linkage between NDM-1-positive isolates [85,88].

Intriguingly, the *bla*_NDM-1_ common region may have resulted in the duplication of *bla*_OXA-10_, *aadA1*, *qacEdelta1*, and *sul1*. In 2011, duplication had been observed for the non-ESBL *bla*_SHV-11_ gene in different strains of *K*. *pneumoniae*, which was linked to a 16-fold higher level of resistance to amoxicillin [89]. More recently, multiple copies of resistance genes (*bla*_SHV-12_, *bla*_OXA-9_, and *bla*_TEM-1_) were reported in a *K*. *pneumoniae* ST11 strain isolated in 2013 in South Korea [90]. When mapping reads to a specific genomic region, the number of aligned reads should be proportional to the number of times the region is present in the isolate [91]. In our simple estimation of relative coverage in CFSAN044566 and CFSAN044568, the four genes (*bla*_OXA-10_, *aadA1*, *qacEdelta1*, and *sul1*) showed a higher coverage when compared to that of *bla*_NDM-1_. Future research with long-read sequencing will be necessary to confirm gene duplication, in addition to closing putative plasmids and determining if they match a previously described plasmid or represent a new variant.

Considering the identification of high-risk clones with extended multidrug resistance, gene duplication, and high prevalence of *bla*_NDM-1_ and *bla*_OXA-48_ genes, sometimes concurrently, our findings highlight the serious challenges posed by MDR *K*. *pneumoniae* and underscores the importance of implementing worldwide surveillance for antimicrobial resistance. Additionally, the numerous potential transmission routes at the human-animal-environment interface stresses the importance of a One Health approach [14] for effective surveillance, control and prevention. The presence of about 5,200 isolates of *K*. *pneumoniae* in a shared database such as NCBI Pathogen Detection should help track the global spread of these deadly pathogens, but it is only as good as the data deposited—a shared responsibility of the clinical research community [92]. The application of WGS to molecular epidemiology studies could provide a better understanding of the worldwide dissemination of MDR isolates and offer a robust surveillance tool that will be useful in detecting and characterizing both existing and emerging threats.

## Disclaimer

The findings and conclusions in this report are those of the author(s) and do not necessarily represent the official position of the U.S. Food & Drug Administration and U.S. Centers for Disease Control and Prevention. The mention of company names or products does not constitute endorsement by the FDA or CDC.

## Supporting information

S1 Table Modifications and amino acid alterations in different genes associated with colistin resistance. Susceptibility or resistance to colistin is indicated with S or R, respectively. The genome sequence for *K*. *pneumoniae* HS11286 was used as a reference (GenBank assembly accession GCA_000240185.2).
(XLSX)

S2 Table Phage profile of CFSAN044572 and CFSAN044573 as determined by PHASTER (PHAge Search Tool Enhanced Release—http://phaster.ca). The tool identified 1 incomplete, 2 questionable, and 2 complete phages.
(XLSX)
